# Decoy peptide targeted to Toll-IL-1R domain inhibits LPS and TLR4-active metabolite morphine-3 glucuronide sensitization of sensory neurons

**DOI:** 10.1038/s41598-017-03447-9

**Published:** 2017-06-16

**Authors:** Yohance M. Allette, Youngsook Kim, Aaron L. Randolph, Jared A. Smith, Matthew S. Ripsch, Fletcher A. White

**Affiliations:** 1 Medical Science Training Program, Indiana University School of Medicine, Indianapolis, IN USA; 2 Department of Anesthesia, Indiana University School of Medicine, Indianapolis, IN 46202 USA; 3 Research and Development Services, Richard L. Roudebush VA Medical Center, Indianapolis, IN 46202 USA

## Abstract

Accumulating evidence indicates that Toll-like receptor (TLR) signaling adapter protein interactions with Toll/Interleukin-1 Receptor (TIR) domains present in sensory neurons may modulate neuropathic pain states. Following ligand interaction with TLRs, TIR serves to both initiate intracellular signaling and facilitate recruitment of signaling adapter proteins to the intracytoplasmic domain. Although TLR TIR is central to a number of TLR signaling cascades, its role in sensory neurons is poorly understood. In this study we investigated the degree to which TLR TIR decoy peptide modified to include a TAT sequence (Trans-Activator of Transcription gene in HIV; TAT-4BB) affected LPS-induced intracellular calcium flux and excitation in sensory neurons, and behavioral changes due to TLR4 active metabolite, morphine-3-glucuronide (M3G) exposure *in vivo*. TAT-4BB inhibited LPS-induced calcium changes in a majority of sensory neurons and decreased LPS-dependent neuronal excitability in small diameter neurons. Acute systemic administration of the TAT-4BB reversed M3G-induced tactile allodynia in a dose-dependent manner but did not affect motor activity, anxiety or responses to noxious thermal stimulus. These data suggest that targeting TLR TIR domains may provide novel pharmacological targets to reduce or reverse TLR4-dependent pain behavior in the rodent.

## Introduction

Activation of Toll-like receptor 4 (TLR4) using lipopolysaccharide (LPS) is known to induce pro-inflammatory cytokine production by monocytes and macrophages. TLR4 is not restricted to immune cells as numerous primary afferent sensory neurons exhibit functional TLR4. Endogenous and exogenous TLR4 agonists including the opioid receptor-inactive morphine metabolite morphine-3-glucuronide (M3G) produce rapid increases in neuronal calcium flux synonymous with elevated states of excitability and potentiation of voltage-gated sodium current in nociceptive sensory neurons^1–7^.

Little is known regarding neuronal TLR4 signaling in rodent neurons. TLR4 is known to be comprised of an extracellular domain with multiple leucine-rich repeats, a single transmembrane helix, and an intracellular region approximately 150 amino acids in size composed primarily of the Toll/IL-1R (TIR) resistance domain^8, 9^. The four TIR-containing adapter proteins, myeloid differentiation primary response gene 88 (MyD88), TIR domain-containing adapter protein (TIRAP), TIR domain-containing adaptor inducing IFN- β (TRIF) and TRIF–related adaptor molecule (TRAM), are responsible for the propagation of signal to downstream targets in a number of cell types^10–12^. In monocytes, interaction of LPS with TLR4 elicits cellular signaling through either MyD88-dependent or MyD88-independent pathways.

The MyD88-dependent pathway, common to the TLR family of receptors, serves to activate NFκB and elicit production of tumor necrosis factor alpha (TNFα) and interleukin-1beta (IL-1β)^13, 14^. Demonstration of the importance of these interactions is best illustrated by mice with a TLR4 point mutation in the MyD88 TIR domain which renders mice LPS-resistant^15^. MyD88 signaling in the nervous system is not limited to TLRs as the pro-inflammatory actions of interleukin-1 type 1 receptor ([IL1R1] also known as CD121a [Cluster of Differentiation 121a]) requires MyD88-mediated activation^16^ to signal the presence of ongoing tissue inflammation^17^. In contrast, a MyD88-independent pathway is associated with only TLR3 and TLR4 and initiates the specific TRIF/interferon-β pathway^18, 19^.

Adapter recruitment by TIR-MyD88 may be important for governing the TLR response in the injured nervous system. Recent findings suggest that mice with a deficit in functional MyD88^20–22^ or a null mutation combination of both MyD88 and TRIF exhibit partial reversal of injury-induced behavioral hypersensitivity which was not observed in TRIF null mice^21^. Though these studies reveal the necessity of TLR signaling^21, 22^, the activation of DRG sensory neurons using LPS appears to be independent of TRIF^23^.

One manner in which to identify the importance of TLR protein-protein interactions in the peripheral nervous system is to target the structurally-conserved BB-loop dependent TIR-TIR signaling^13, 24^. Initial studies by Toshchakov and colleagues screened libraries of peptides which mapped the entire surface of TLR4 TIR^24^. Decoy peptides were then tested for their ability to bind to different portions of TLR4 TIR domain and inhibit LPS-induced production of cytokines using peritoneal macrophages derived from mice. Subsequent findings determined that the decoy peptide 4BB strongly binds to TLR4 TIR^24, 25^ and inhibited LPS-associated cytokine production in macrophages^24^.

Based on the ability of decoy peptides to interfere with LPS-induced proinflammatory cytokine production by macrophages, we observed that 4BB decoy peptide modified to include TAT (TAT-4BB) could diminish LPS-induced changes in intracellular calcium [Ca^2+^]_i_ and excitability in acutely dissociated sensory neurons. We further show that rapid induction of mechanical allodynia by the TLR4-active metabolite, M3G, could be inhibited in the presence of TAT-4BB but basal pain response to thermal stimulus was unaffected.

## Methods

### Animals

A total of 46 pathogen-free adult Sprague-Dawley rats from Harlan Laboratories (average weight of 150–200 g) were randomly assigned to treatment groups used for the described experiments. A total of 16 adult female pathogen-free C57BL/6J mice from Harlan Laboratories were used (average weight of 25–30 g). Experiments were designed to minimize animal discomfort and the amount of animals needed for statistical evaluation. All behavioral studies were conducted during the 12 hour light cycle; controlled animal housing was maintained at a temperature of 23 °C (with a deviation of 3 °C) with 12 hour light and dark cycles daily. Mice were housed in groups by littermates. All animals were supplied with standard rodent chow and autoclaved tap water. The Institutional Animal Care and Use Committee (IACUC) of the Indiana University School of Medicine approved all disclosed experiments per protocol #10492. Animal procedures adhered to guidelines outlined within the Guide for and Care and Use of Laboratory Animals of the National Institutes of Health, with ethical guidelines outlined by the International Association for the Study of Pain. All behavioral data analysis was blinded.

### Reagents

For each experiment, all reagents were prepared immediately in buffer prior to the procedure. Biologically reactive O5:55 serotype lipopolysaccharide (LPS) from Sigma (St. Louis, MO) was reconstituted in deionized water. NIH/NIDA Drug Supply Program provided morphine-3-β-D-glucoronide (M3G). Acute dissociation culture was media made of DMEM, Ham’s F-12 mixture, N2 (Life Technologies Corp.), penicillin (100 μg/mL), streptomycin (100 U/mL), and supplemented with 10% fetal bovine serum. The 4BB decoy peptide sequence (LHYRDFIPGVAIAA) was modified to include the TAT transduction domain of human immunodeficiency virus-1 segment (YGRKKRRQRRR) in order to increase membrane permeability in the rodent and neuronal cell cultures^26^. Peptide order was the TAT peptide sequence followed by the 4BB peptide sequence. A control peptide with TAT modification used a scrambled amino acid sequence (SLHGRGDPMEAFII)^24^. Tissue and cell distribution studies utilized fluorescent FITC-labeled TAT peptide alone or in conjunction with the 4BB peptide. All peptide preparations were diluted in saline solution. For intraperitoneal injections, a concentration of 10 mg/kg was used for all 4BB preparations. The *in vitro* applications of FITC-TAT-4BB used a concentration of 15 μM. Vehicle controls consisted of saline for all experiments. All peptides used for outlined experiments were created by GenScript utilizing their peptide synthesis service.

### Tissue processing for FITC-TAT-4BB tissue

Animals were injected with 10 mg/kg FITC-TAT-4BB and sacrificed 15 minutes later. The animals were euthanized and perfused transcardially with a saline solution followed by 4% paraformaldehyde fixative. The fixed tissue was cryoprotected with 30% sucrose then sectioned via the Leica Biosystems 3050 research cryostat (Buffalo Grove, IL) at a thickness of 15 μm. Before imaging, slides were stained with 4′ 6-diamidino-2-phenylindole (DAPI) for an exposure time of 5 minutes. An intensified charged coupled device camera (CoolSnap HQ2, Photometrics; Tucson AZ) connected to a Nikon microscope using Nikon Elements Software (Nikon Eclipse Ti, Nikon Instruments Incorporated, Melville, NY) was used for image acquisition.

### Preparation of acutely dissociated dorsal root ganglion neuron

Dorsal root ganglia L4 – L6 were used as the source for a nociceptive primary cell culture population. The DRG tissues were dissected and collected immediately post mortem^27^. The collected samples were initially dissociated using a solution of DMEM, neutral protease, and collagenase (Worthington Biochemical) at a temperature of 37 °C on a rocker at a low setting for 40 minutes. The tissue was further dissociated by mechanical trituration and plated on poly-L lysine and laminin coated coverslips (BD Biosciences) in a culture media of 1 mg/mL bovine serum albumin and trypsin inhibitor (Worthington Biochemical). Following plating, cells were incubated for 2–3 hours, and then another 1 mL of culture media was adding to each well. The cells were then incubated overnight for 12–15 hours at a temperature of 37 °C with 5% CO_2_ to improve adhesion to coverslips prior to experimentation. Some sensory neurons were exposed to FITC-TAT-4BB for 15 minutes using 15 μM solution.

### Ca^2+^ imaging in acutely dissociated dorsal root ganglion neurons

Dissociated DRG cells were exposed to Fura 2-AM at 3 mM (Invitrogen) for at least 25 minutes at room temperature. Intracellular calcium changes were monitored in a balanced salt solution (BSS). BSS was composed of NaCl 140 mM, Hepes 10 mM, glucose 10 mM, KCl 5 mM, CaCl_2_ 2 mM, and MgCl_2_ 1 mM. Following Fura 2-AM exposure, coverslips were rinsed a total of three times in BSS. The changes in intracellular calcium (Ca^2+^) were observed and recorded with digital video microflurometry using an intensified CCD camera coupled to a Nikon Eclipse TE2000-U microscope with Nikon Elements® software. Using a 150 W xenon arc lamp, coverslips were illuminated – the 340/380 nm excitation wavelengths of Fura 2-AM were selected by Lambda DG5 plus illumination system (Sutter Instruments, Novato CA). Prior to treatment administration, sterile BSS was applied, and any cells showing a response of any kind to buffer alone were excluded from data collection.

The calcium imaging experiments were modified from the previous application (Allette *et al*.^6^). A minimum of three minutes between treatments allowed to make sure that the signal re-established to the baseline level immediately prior to the treatment. Following a baseline measurement of intracellular calcium changes for 1 minute, Ca^2+^ influx was initiated by the addition of either LPS 1–2 μg/mL or M3G 3 μM. Both LPS and M3G treatments were diluted in Tyrode’s solution. Cells were first exposed to TAT-4BB at a concentration of 15 μM before administration of the second LPS treatment.

All traces recorded from calcium imaging were independently analyzed by two different researchers. The preset criteria for data collection included a 340 nm/380 nm fluorescence ratio that must exceed the baseline value preceding treatment administration by 10%, and that two independent data analysts agreed on all recorded cell responses used in cell counts.

### Electrophysiology

Using the inverted microscope (Nikon Eclipse Ti; Nikon Instruments Inc. Melville, NY) sharp electrode intracellular recordings from primary afferent neurons were collected at room temperature. The primary afferent neurons were used 12–18 hours following acute dissociation. Micropiptettes for recording were made on a Flaming/Brown micropipette puller (P-98 Sutter Instruments) and made of borosilicate glass (Word Precision Instruments; Sarasota FL). Micropipettes were filled with a 1.0 M KCl solution (40–80 MΩ impedance) prior to recording. To bridge-balance the micropipette resistance, a current injection of −0.1 nA was used. A micromanipulator (Newport Corporation; Irvine, CA) was used for exact positioning of the micropipettes. The recording chamber was perfused with a saline solution (NaCL 120 mM, Hepes 10 mM, glucose 10 mM, KCl 3 mM, CaCl_2_ 1 mM, MgCl_2_ 1 mM).

All neurons were classified by soma diameter (large ≥45 μm, medium 30–45 μm, small ≤30 μm). Only neurons exhibiting a resting membrane potential greater that −45mV were accepted for study. All neurons used in study were measured for a 1 minute period under without any external treatments or stimulus. Both small and medium sized neurons were measured for neuronal excitability by injection of current pulses directly into the soma at intervals of 30 seconds, with injections consisting of 1 second pulses (Due *et al*.^5^). Current was adjusted accordingly to ensure firing of 1–2 action potentials per current injection in baseline conditions. Electrophysiological recordings utilized continuous current-clamp in bridge mode, and used an AxoClamp-2B amplifier. Data were stored through the Digidata 1322 A interface. Data analysis was completed offline using the pClamp 9 software from Axon Instruments.

Following 3 control current injections, LPS at 1–2 μg/mL was applied and current injections continued every 30 seconds. Exposure to the decoy peptide at a concentration of 15 μM prior to application of LPS at 1–2 μg/mL was experiment-dependent. Neuronal excitability was measured as the number of action potentials elicited per current pulse both prior to and following the application of LPS. In the case of increased neuronal excitability following LPS administration, either the decoy peptide or vehicle treatment was applied to determine the reversal of the LPS-driven neuronal excitation.

### Mechanical Withdrawal Threshold Measurements

A minimum 2 day period was utilized for the habituation of all animals to the behavioral testing chambers. Rodents were randomly assigned to either the vehicle or M3G treatment groups. Von Frey filaments measuring 200 μm in diameter were used to elicit responses to mechanical indentation of the plantar aspect of the animal hindpaw^28^. At designated loci on the plantar surface force was applied using filaments (10, 20, 40, 60, 80, 120 mN). Each stimulus lasted approximately 1 second and had a 10–15 second intermission between applications. The incidence of foot withdrawal was recorded as a percentage of six stimuli and the percentage of requisite withdrawal; results were subsequently plotted as a function of force. Optimal TAT-4BB dosing was first determined using small pilot groups, with concentrations of 0.1, 1, and 10 mg/kg. Animals were assessed both prior to treatment, and 1 and 4 hours status post treatment. Individual testers blinded to decoy peptides completed all behavioral assessments and data collection. A total of five independent animal groups consisting of 6–8 animals, were used for behavior experiments.

### Foot Withdrawal to Plantar Thermal Stimulus

The Hargreaves’ plantar test apparatus (Ugo Basile; Varese, Italy) was used to assess the effects of nocifensive behavior of uninjured animals following noxious thermal stimulation in the absence and presence of the decoy peptides. The apparatus has a glass floor of 2mm thickness kept at a temperature of 22.5–23.5 °C, and contains a mobile infared heat generator. The heat generator aperture was focused on the animal hindpaw through the glass floor. The latency to withdrawal (i.e. reaction time) was recorded following the activation of the heat generator. If a shortening of the withdrawal latency was observed, this was considered thermal hyperalgesia.

Following animal habituation to the apparatus and environment, measurements of withdrawal latency were repeated a total of 4 times at 5 minute intervals on each paw. The setting of the heat generator was at IR = 70, and the initial pair of data for each paw was not used in data analysis; only the average of the three remaining data pairs were utilized. A total of three naïve animals were assessed with the decoy peptide.

### Open-field test as a measure of anxiety-associated behavior

Female mice were used for behavioral analyses. All behavioral testing performed in female mice was completed prior to the start of the estrous cycle. Prior to any behavioral testing, the mice were brought into the procedure room and allowed to habituate to the room for 30 minutes. Animal numbers per group for behavioral tests are indicated in figures. All behavioral analyses were done by observers who were blinded to drug condition. The open-field arena covered an area of 40 cm × 40 cm, with 40 cm high walls. The outside walls of the chamber were opaque. During this one-time test, an animal was placed in the empty plexiglass arena and allowed to explore for 10 minutes. The position of the mouse was tracked using AnyMaze (Stoelting, Wood Dale, IL). Distance traveled, average speed, time spent in inner ring and total time immobile were analyzed per 10-minute video.

### Statistics

All enclosed data are presented as group mean ± SEM following statistical analysis using GraphPad Software (LaJolla, CA); a p-value of <0.05 was set as the threshold for statistical significance. All baseline data for tactile tests were recorded prior to experimental procedures (i.e. drug and vehicle dosing to animals). Repeated measures of ANOVA (analyses of variance) followed by post-hoc Tukey’s multiple comparison test were used to assess data from each treatment group compared to respective baseline values. Both tactile and thermal threshold time course were analyzed, and plotted as the mean ± SEM compared to time after treatment. Open field experiment data was analyzed by two-tailed unpaired Student’s t-test. The chi-square test with Yates correction was used for assessing the statistical significance of the differences in calcium response for both capsaicin sensitive and non-capsaicin sensitive neurons.

## Results

### Distribution of the cell-penetrating protein trans-activator protein (TAT) *in vivo*, and TLR4/TIR-specific decoy peptide 4BB *in vitro*

**Figure 1:**
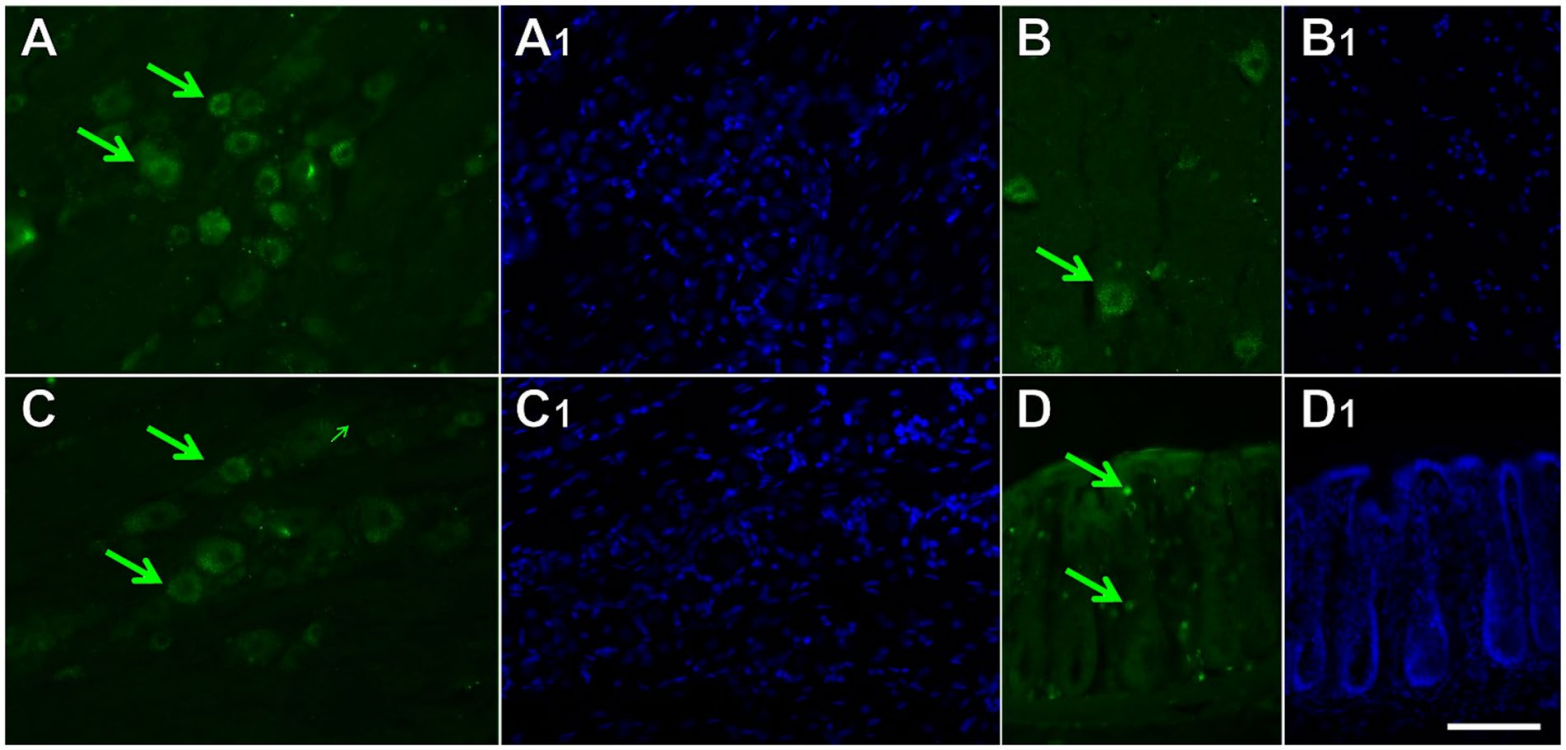
Cryosections of dorsal root ganglion (DRG), trigeminal ganglion (TRG), ventral spinal cord and colon in the rat thirty minutes after intraperitoneal injection of FITC-TAT-4BB. (**A**) FITC-TAT-4BB present in a number of primary afferent sensory neurons in lumbar DRG (green arrows). (**B**) FITC-TAT-4BB present in a number of motor neurons in ventral spinal cord (green arrows). (**C**) FITC-TAT-4BB present in a number of primary afferent sensory neurons in TRG (green arrows). (**D**) FITC-TAT-4BB present in a number of presumptive immune cells based on size and location (green arrows). Nuclei of all cells are stained with DAPI (A_1_, B_1_, C_1_, D_1_ blue). Scale bar is 100 μm.

**Figure 2:**
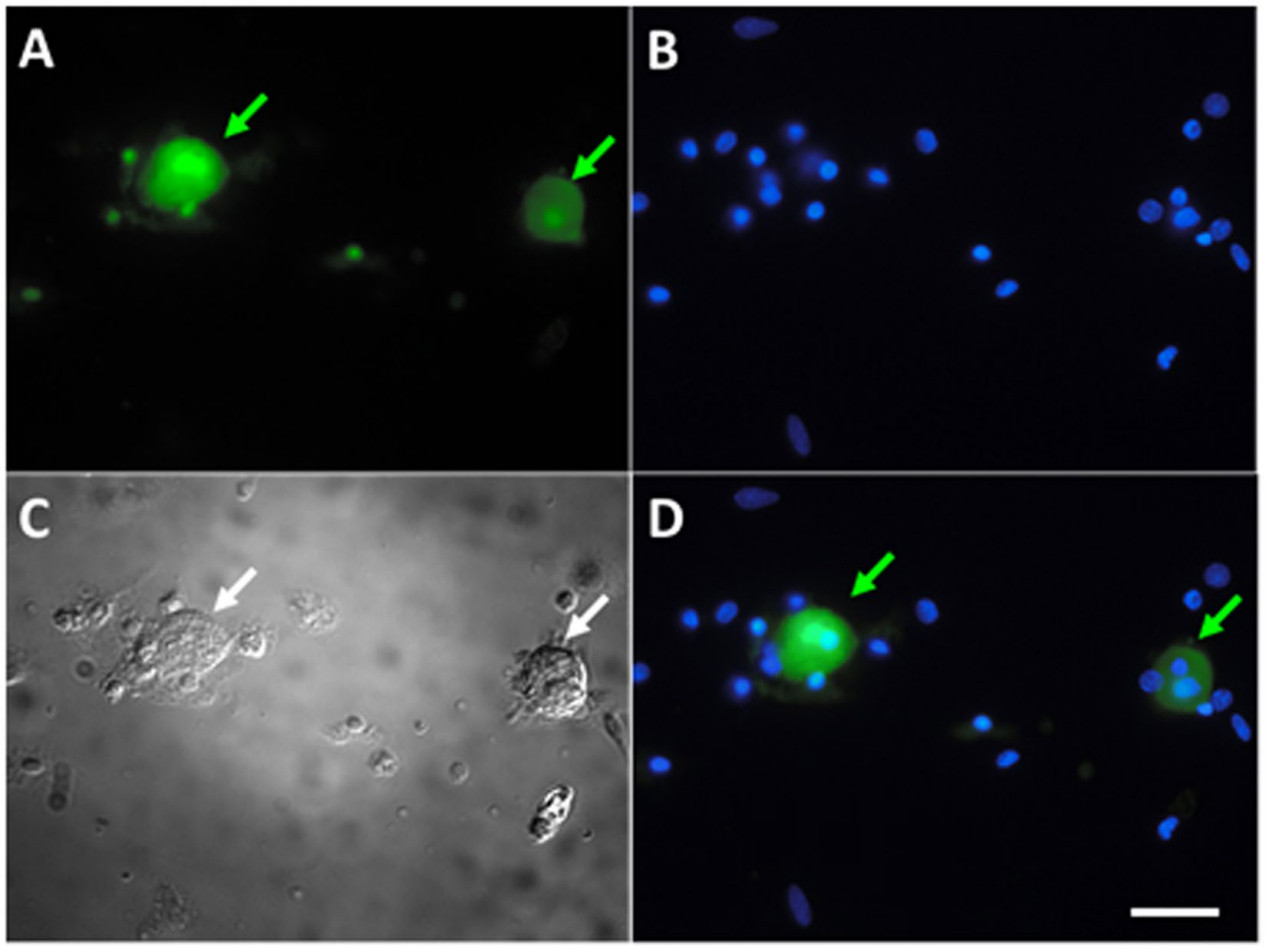
FITC-TAT-4BB predominately accumulates in acutely dissociated neurons (**A**, green arrows). Nuclei of all cells are stained with DAPI (**B**, blue). Bright field of representative acutely dissociated cells (**C**). Merged images are shown in (**D**). Scale bar is 20 μm.

Recent studies targeting the surface-exposed segments of TLR4 TIR domain proteins with decoy peptides demonstrate effective inhibition of LPS-mediated macrophage signaling using a cell-permeating protein in the form of the Antennapedia homeodomain sequence^24^. Inclusion of the Antennapedia homeodomain allows peptides to cross the blood brain barrier but the tagged peptide is selectively taken up by a subpopulation of neurons, suggesting a restricted penetrance of some cell layers or tissue^29^. Another cell-penetrating protein, HIV trans-activator protein (TAT) has also proven to be effective in transporting large and small molecules across cellular membranes into a larger population of neurons^26, 30^. To assay the degree to which TAT efficiently distributes to cells present in tissues of interest, we intraperitoneally injected TAT-4BB labelled at the N-terminus with FITC (FITC-TAT-4BB) into rats. Systemic exposure to FITC-TAT-4BB produced fluorescent label in almost all neurons in the trigeminal and dorsal root ganglia (Fig. 1A and C), some ventral motor neurons in the spinal cord (Fig. 1B) and numerous non-neuronal cells in the gastrointestinal tissue (Fig. 1D).Distribution of FITC-TAT-4BB following *in vitro* exposure produced robust penetration of sensory neurons (based on morphology and diameter) but was absent in non-neuronal cells (Fig. 2).

### TLR4/TIR decoy peptides decrease M3G and LPS-induced Ca^2+^ mobilization in dissociated sensory neurons

We next sought to determine whether LPS and M3G can directly excite DRG neurons by examining their ability to elicit calcium signals. Activation of sensory neurons using LPS or the TLR4-active morphine metabolite, M3G, results in detectable changes in cytoplasmic calcium ([Ca^2+^]_i_)^6, 31, 32^ and the use of a TLR4 small molecule inhibitor effectively blocks [Ca^2+^]_i_ in these same sensory neurons^4, 6^. Herein, cultured neurons rapidly responded to LPS and to M3G, as indicated by increased intracellular calcium levels, suggesting that DRG neurons express TLR4. The order of exposure to the LPS followed by M3G induced more responses than M3G followed by LPS (Table 1).

**Table 1:** Calcium imaging of DRG neurons.

Order of Protein Tested	^#^Neuronal responses/total ^#^Of neurons, (%)	^#^Neuronal responses to both LPS and M3G/total ^#^Of neurons, (%)	^#^Animals per assay
(*1*) *LPS*	26 of 58 (44%)		4
(*2*) *M3G*	10 of 58 (17%)	38.5%*	4
(*1*) *M3G*	9 of 64 (14%)		4
(*2*) *LPS*	19 of 64 (29%)	89%*	4

*p < 0.05, Chi-square with Yates correction.

**Figure 3:**
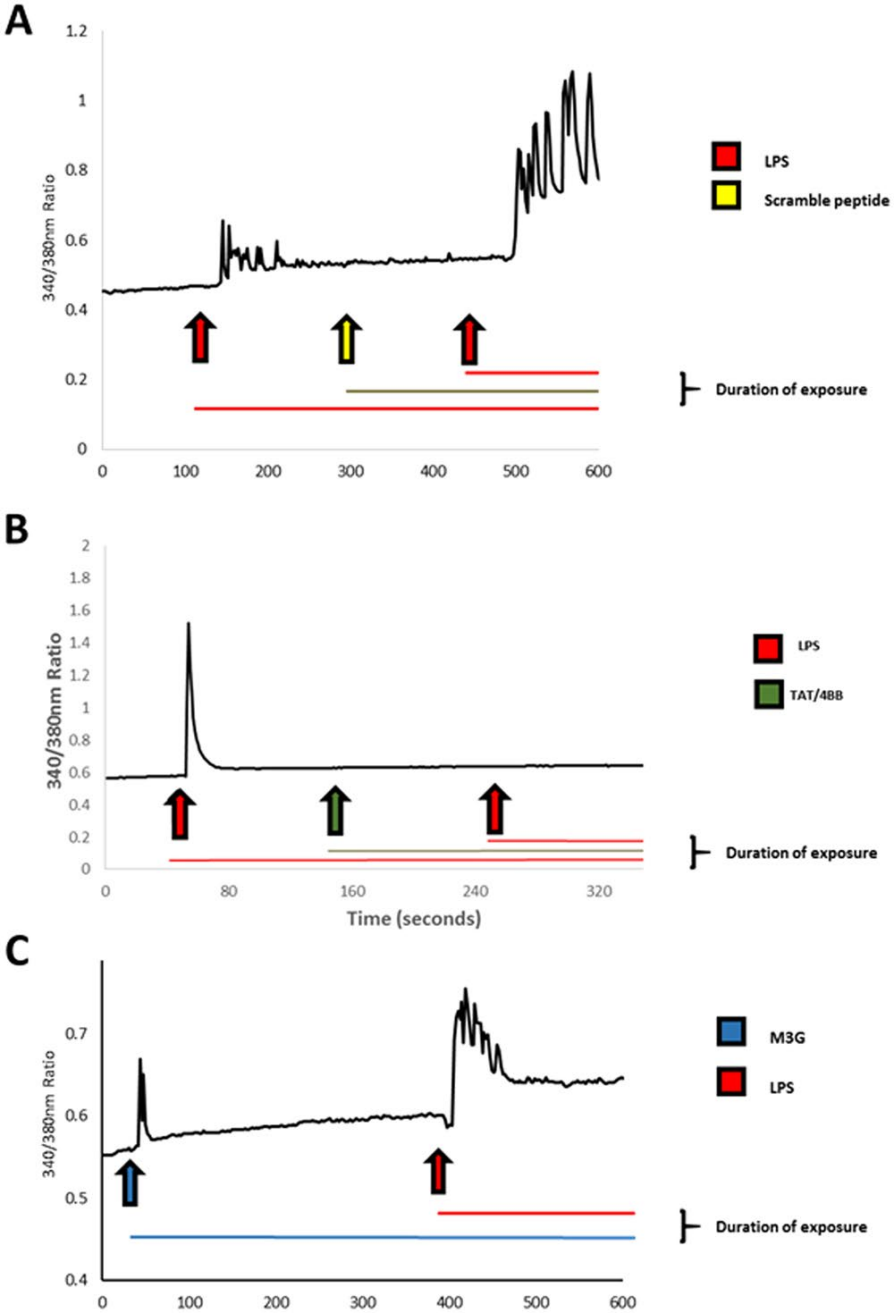
Lipopolysaccharide (LPS) and morphine-3-glucuronide (M3G) increases in calcium signaling in cultured dorsal root ganglion (DRG) neurons can be prevented by exposure to TAT-4BB. Ca^2+^ imaging was performed on acutely dissociated adult sensory neurons using the ratiometric Ca^2+^- sensitive dye, FURA-2AM. (**A**) Selected trace depicts neuron response to LPS followed by wash out of the well with room-temperature bath solution. Cells were then exposed to control scramble peptide followed by LPS exposure. Control peptide did not alter LPS-induced event. (**B**) Selected trace depicts neuron response to LPS followed by wash out of the well with room-temperature bath solution. Cells were then exposed to TAT-4BB peptide followed by LPS exposure. Second LPS exposure failed to elicit change in Ca^2+^-flux. (**C**) Selected trace depicts neuron response to M3G followed by wash out with bath solution. Cells were then exposed to LPS which elicited a second Ca^2+^-flux.

To determine the effect of TAT-4BB in the presence of LPS, we first demonstrated that none of the cells which were affected by the presence of the TAT-control peptide (n = 4 animals; n = 25 cells) (Fig. 3A). LPS responsive cells were then exposed to TAT-control peptide or TAT-4BB (15 μM) followed by a second exposure to LPS. A large number of LPS-responsive neurons (71%) were desensitized to the second exposure of LPS following TAT-4BB (n = 4 animals; 38 total cells) (Fig. 3B). Previous observations indicate that LPS and M3G exposure produce increases in [Ca^2+^]_i_ in the similar neuron populations and can be inhibited by a small molecule inhibitor of TLR4 (Due *et al*.^4^). Based on these observations, we demonstrate that most M3G responsive sensory neurons also respond to LPS exposure (89%; n = 9 cells) (Fig. 3C).

### TAT-4BB decoy peptide diminishes inward current facilitation in nociceptive neurons

TLR4 agonists such as LPS have been shown to elicit inward current in nociceptive sensory neurons and increase the excitability of sensory neurons^1, 3, 4^. These agonists also elicit activation of TLR4-mediated excitation in nociceptive neurons increases density in a number of voltage-gated sodium currents^4, 5^. To determine the degree to which the decoy peptide TAT-4BB inhibited LPS-induced neuronal excitation, we examined neuronal response using sharp electrodes in current clamp mode.

**Figure 4:**
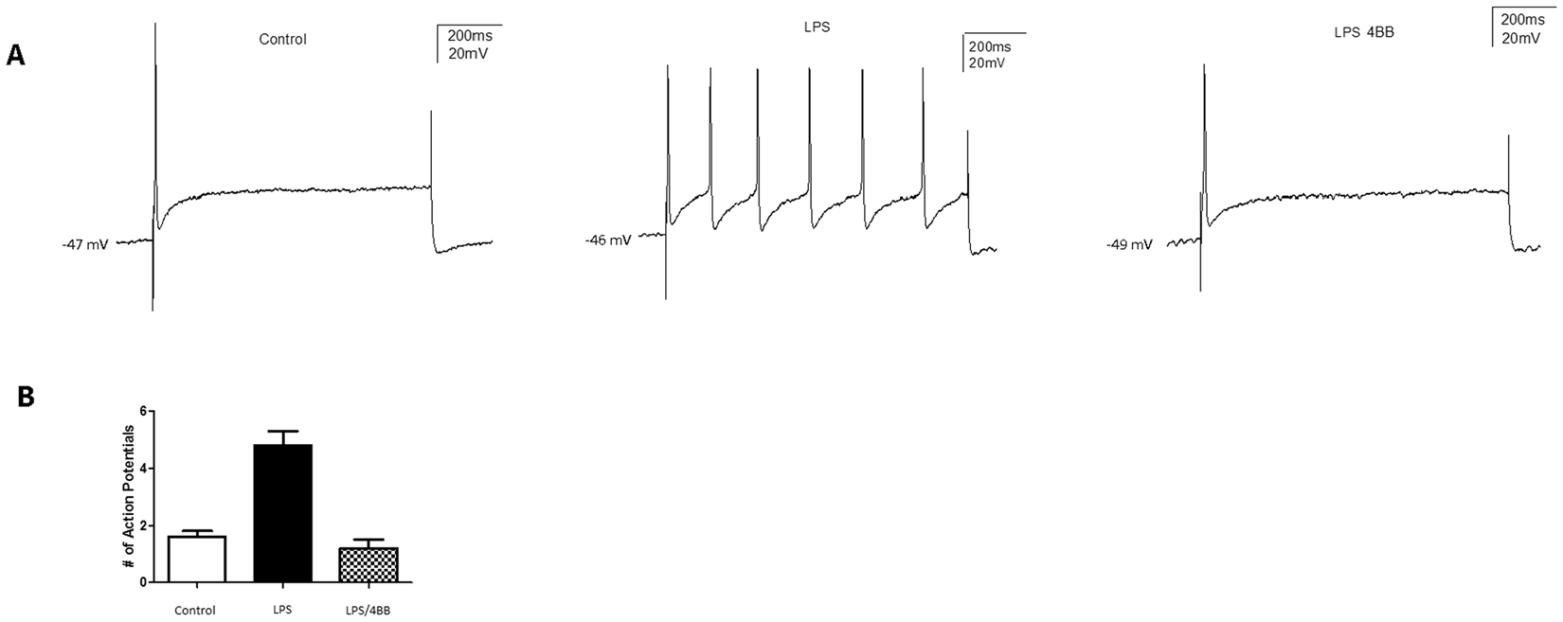
Lipopolysaccharide (LPS) increases in nociceptive neuron excitability is reversed by TAT-4BB. (**A**) Current clamp recordings were performed on small-to-medium (>30 μm–>40 μm) diameter lumbar 4–5 dorsal root ganglia (DRG) neurons from naive rats. Firing of 1–2 action potentials was elicited by a 1 second depolarizing current injection (ranging from 0.1 to 2.0 nA depending on the cell) every 30 seconds. Representative recordings demonstrating that application of LPS and the subsequent increase in elicited action potentials in DRG sensory neurons can be reversed by administration of the 4BB MyD88 decoy peptide (n = 9 cells). (**B**) Group data showing that LPS caused a significant increase in DRG AP firing which is reversed by addition of TAT-4BB. Values are based on an n = 9 cells ∗ = p < 0.01.

Repeated current pulse combined with LPS administration produced a significant increase in the excitability of small diameter neurons when compared to baseline levels, though less than 27% of neurons responded to LPS (n = 13 of 49 cells). We also observed 1.6 ± 0.2 APs in cells under control conditions compared to 4.8 ± 0.6 APs in cells subjected to LPS (cell n = 9 cells; animal n = 5) (Fig. 4A). Subsequent treatment with TAT-4BB completely blocked LPS-dependent sensitization in all sensory neurons that responded to LPS (1.2 ± 0.2 APs for TAT-4BB, n = 9 cells; F = 41.82, p < 0.05; Dunnett’s multiple comparison test, p < 0.05) (Fig. 4B). Exposure of LPS responsive neurons to control peptide did not alter LPS-induced neuronal sensitization (data not shown). These studies show that in addition to TAT-4BB reducing LPS-induced [Ca^2+^]_i_ in sensory neurons, TAT-4BB suppresses sensitization of sensory neurons in the presence of the TLR4 ligand LPS.

### Pretreatment of rodents with TAT-4BB prevents the rapid induction of tactile hyperalgesia due to systemic morphine-3-glucuronide administration

**Figure 5:**
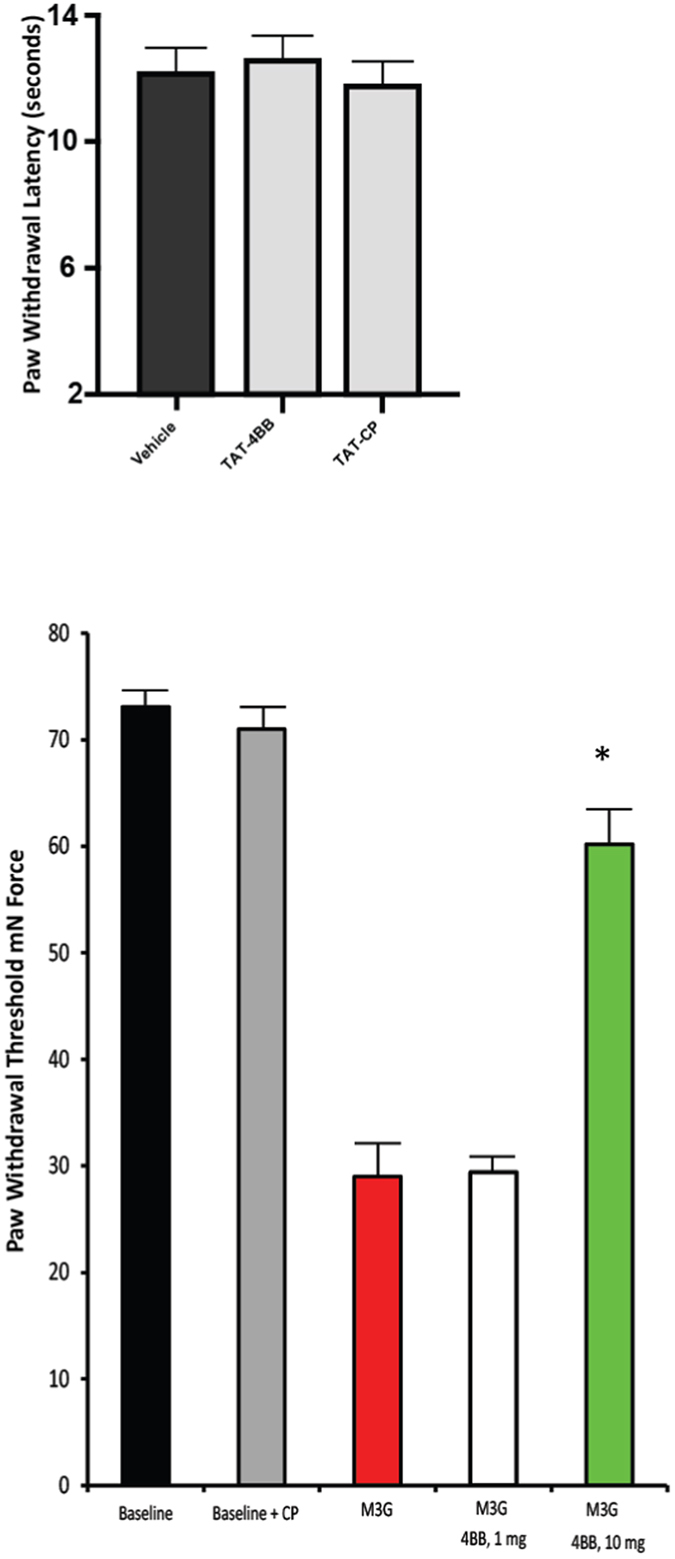
Effects of cell-permeable TAT-4BB decoy peptide on thermal sensitivity and tactile allodynia. (**A**) TAT-4BB and TAT-CP was tested for the ability to alter nocifensive behavior in naïve rats. (**B**) TAT-4BB was tested for the ability to prevent sensitivity to tactile stimulus one hour after M3G administration. TAT-4BB potently affected behavioral hypersensitivity at 10 mg/kg but not 1 mg/kg. Values are the mean ± SEM of two groups of 8 rats per time point. (∗ = p < 0.05 TAT-4BB in combination with M3G rats compared to M3G treatment alone).

Systemic M3G administration produces rapid induction of mechanical allodynia but not thermal hyperalgesia in rodents through a TLR4 dependent mechanism^4^. The poor permeability of M3G into brain tissue following intravenous bolus suggests that the behavioral effects of the metabolite are due to drug interactions in the peripheral compartment^33, 34^. Based on the presumptive peripheral effects of intraperitoneal injection of M3G on paw withdrawal threshold (32.4 ± 3.4 mN; *n* = 16), we observe that pretreatment with TAT-4BB (10 mg/kg, i.p.) effectively eliminates changes in paw withdrawal threshold to tactile stimulus when compared with control peptide (10 mg/kg, i.p.; n = 8 per dose, 60.14 ± 4.37 mN; F = 12.81, p < 0.05) (Fig. 5).

### TAT-4BB decoy peptide did not alter open-field behavior

**Figure 6:**
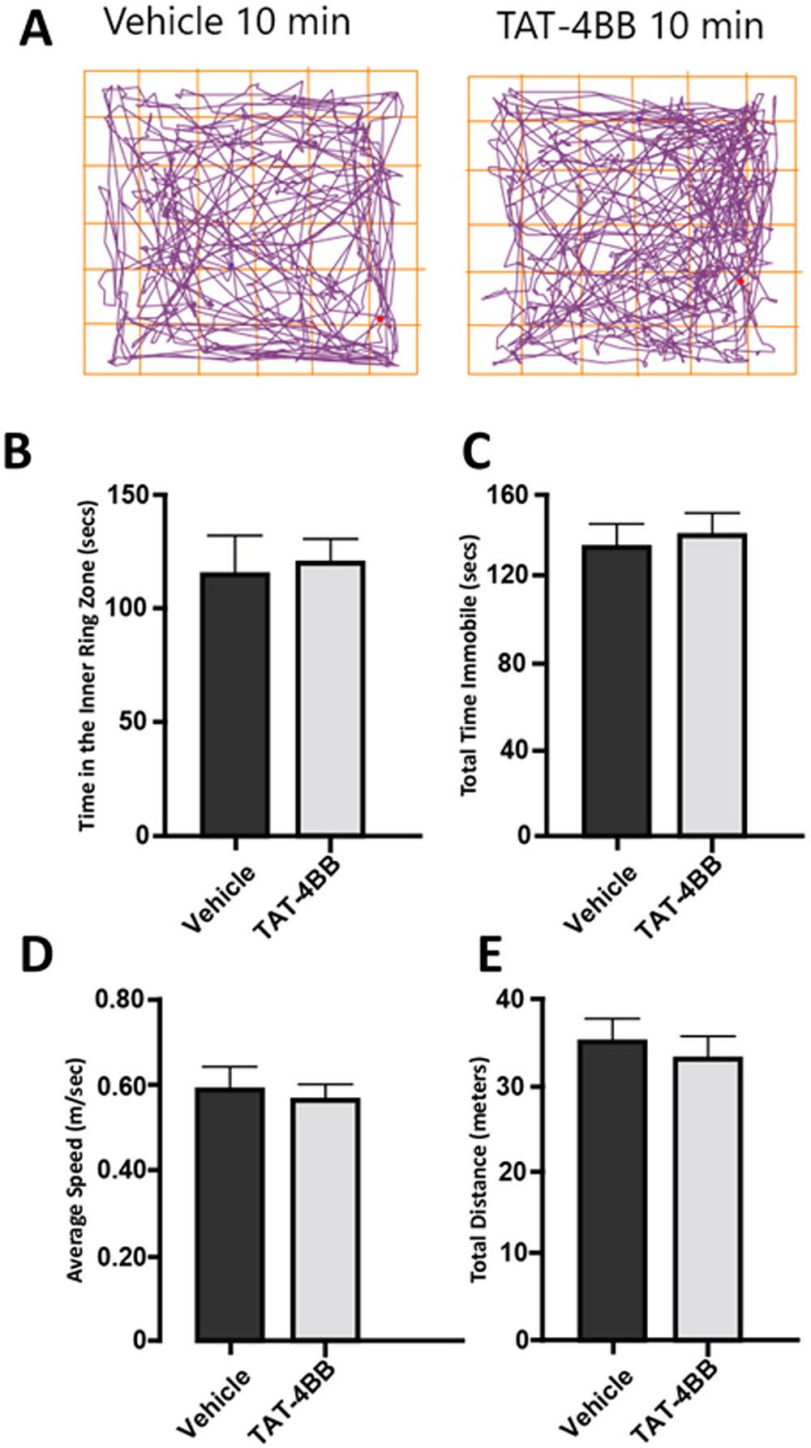
TAT-4BB does not produce deficits in anxiety-like behavior in female mice using open field testing (OFT). (**A**) Representative activity traces for rodents in the OFT following TAT-4BB administration. (**B**) Total time spent in the inner ring zone. (**C**) Total time immobile (**D**) Average speed (**E**) Total distance traveled. Data in both vehicle (n = 5) and decoy peptide (n = 8) reflects means and SEM.

As a measure of possible off-target effects of the decoy peptide we investigated the possible effects of TAT-4BB on anxiety-like behavior in mice. Anxiety in mice reflects a conflict between risk and reward^35^ and can be evaluated in rodents using an accepted test of anxiety-associated behavior, the open-field test (OFT). Rodents with anxiety behavior spend less time exploring the center of the chamber and travel shorter distances^36, 37^. Mice receiving single injections of vehicle (n = 5) or TAT-4BB (n = 8) did not show differences in general locomotor associated behaviors during a either a 5 or 10 minute period of time (distance traveled, *t*_(10)_ = 0.2791, *p* = 0.78; average speed *t*_(10)_ = 0.2681, *p* = 0.7941) in the open-field. In addition, TAT-4BB treated rodents did not show a change in the total time immobile (center time, *t*_(10)_ = 0.4625, *p* = 0.6536) or a preference for spending more time in the inner ring zone of the open-field or compared to vehicle-treated controls (inner ring time, *t*_(10)_ = 0.6793, *p* = 0.5124) (Fig. 6).

## Discussion

Recruitment of the TLR adapter proteins are likely critical to the first intracellular events following neuronal exposure to LPS. Assembly of TLR4 signaling adapter proteins present in nociceptive neurons is followed by rapid changes in action potential discharge, voltage-gated sodium currents, intracellular calcium and activity dependent release of neuropeptides^3, 4, 38^. Though several additional mechanisms may be involved with the neuronal TLR4-mediated events, we demonstrate for the first time that interruption of the TLR TIR-dependent signal transduction using TAT-4BB can inhibit an important upstream pathway for both LPS and M3G-mediated changes in neuronal signaling events. In addition, TAT-4BB can prevent M3G-induced tactile allodynia in rodents.

Ligand binding to TLR4 present on macrophages and microglial cells leads to activation of nuclear factor κB (NFκB) where it initiates the transcription of genes encoding inflammatory cytokines. The activation of this receptor and downstream TLR TIR cell signaling leads to cytokine and chemokine release, though dependent on increases in Ca^2+^, does not produce an immediate increase in [Ca^2+^]_i_^39^. Unlike the relatively slow responses observed in monocytes and microglial cells, activation of neuronal TLR responses are rapid and may contribute to potentiation of neuronal excitability through nonselective cation channels such as TRPV1 or TRPA1^1, 20, 40, 41^, voltage-gated ion channel events^4, 42–44^ or TNF-α mediated events^45^. Similar observations have been documented with another MyD88-dependent cytokine, IL-1β. IL-1β neuronal stimulation can rapidly activate both post-translational changes of proteins and tetrodotoxin-resistant currents in nociceptive cells^17^.

The ability of TLR4 active agonists to elicit rapid intracellular calcium mobilization in sensory neurons predicts that these cells may convey mechanical modalities independent of inflammation states^46^. Though not distinguished herein, transactivation of the TRPV1 channel through TLR4 stimulation has been shown a potential mechanism of nociceptive response^1^. TRPV1-bearing cells may not be only capable of sensing thermal changes as a number of these neurons co-express TRPA1^47, 48^, but also may be cross-sensitized by the presence of TRPA1 agonists^49^. Similar TLR agonist-dependent actions have been described for bacterial N-formylated peptides and the pore-forming toxin α-haemolysin reflecting neuronal TLR2 signaling^44^. The degree to which TAT-4BB influences neuronal TLR2 is unknown but mutations within the BB loop and associated TLR TIR domain is critical for inhibiting TLR2 activation in *Staphylococcus aureus* lipoteichoic acid- and Pam3CSK4-treated macrophages^50^. Though sensory neurons are known to exhibit functional receptors for TLR5 and TLR7^41, 51, 52^, less is known regarding the role of the BB-loop of the TIR domain for TLR5^53^ or TLR7 signaling^54^.

There is a distinct possibility that ion channels may be associated with TLR4 activation including voltage-gated sodium channels. It is known that TLR4 agonists such as the M3G elicits a substantial increase in the current density for voltage-gated sodium channels (NaV), NaV1.6, NaV1.7 and NaV1.9, but not NaV1.8^4^. Increased firing of these channels may contribute to opioid-induced tactile allodynia^4^. These opioid metabolite-induced changes in neuronal excitability can be pharmacologically inhibited by the state-dependent sodium channel blocker, carbamazepine^5^.

Although there is some question regarding the ability of M3G to interact with MD2 using *in silico* approaches^55^ or to elicit macrophage activation *in vitro* ^56^, it appears that M3G elicits consistent TLR4 activation and pro-inflammatory cytokine production in morphine-treated mice^34^. TLR4 activation effects in non-neuronal cell types may also depend on NaV channels. Most notably, phagocytic activity, migrational activity, and systemic inflammatory responses by macrophages and microglia can be modulated by the activity of NaV1.6 but not Nav1.7, Nav1.8, or Nav1.9^57, 58^. However, the sodium ion channel properties and subsequent downstream signaling through p38/mitogen-activated protein kinases or nuclear factor κB (NFκB) necessary for these proinflammatory events are largely unknown^59, 60^.

## Conclusion

In summary, our experiments suggest a role of TLR-TIR signaling in nociceptor sensitization following LPS or M3G. This nociceptor sensitization can be suppressed using TAT-4BB in primary afferent sensory neurons. Further research is required to understand the relative importance of the TLR TIR signaling downstream of the receptor activation in primary afferent sensory neurons and the potential influence on neuronal ion channels.

### Declarations

All animal related experiments were approved by the Institutional Animal Care and Use Committee of Indiana University School of Medicine. All procedures were conducted in accordance with the Guide for Care and Use of Laboratory Animals published by the National Institutes of Health and the ethical guidelines established by the International Association for the Study of Pain.

### Availability of data and materials

All relevant raw data will be made available upon request.
